# Sodium houttuyfonate affects production of N-acyl homoserine lactone and quorum sensing-regulated genes expression in *Pseudomonas aeruginosa*

**DOI:** 10.3389/fmicb.2014.00635

**Published:** 2014-11-26

**Authors:** Daqiang Wu, Weifeng Huang, Qiangjun Duan, Fang Li, Huijuan Cheng

**Affiliations:** ^1^Laboratory of Microbiology and Immunology, School of Chinese and Western Integrative Medicine, Anhui University of Chinese MedicineHefei, China; ^2^School of Pharmacy, Anhui University of Chinese MedicineHefei, China

**Keywords:** Sodium houttuyfonate, *Pseudomonas aeruginosa*, Quorum sensing, N-acylhomoserine lactone, Las system

## Abstract

Quorum sensing (QS) is a means of cell-to-cell communication that uses diffusible signaling molecules that are sensed by the population to determine population density, thus allowing co-ordinate gene regulation in response to population density. In *Pseudomonas aeruginosa*, production of the QS signaling molecule, *N*-acyl homoserine lactone (AHL), co-ordinates expression of key factors of pathogenesis, including biofilm formation and toxin secretion. It is predicted that the inhibition of AHL sensing would provide an effective clinical treatment to reduce the expression of virulence factors and increase the effectiveness of antimicrobial agents. We previously demonstrated that sodium houttuyfonate (SH), commonly used in traditional Chinese medicine to treat infectious diseases, can effectively inhibit QS-regulated processes, including biofilm formation. Here, using a model system, we demonstrate that SH causes the dose-dependent inhibition of AHL production, through down-regulation of the AHL biosynthesis gene, *lasI*. Addition of SH also resulted in down-regulation of expression of the AHL sensor and transcriptional regulator, LasR, and inhibited the production of the QS-regulated virulence factors, pyocyanin and LasA. These results suggest that the antimicrobial activity of SH may be due to its ability to disrupt QS in *P. aeruginosa*.

## Introduction

The Gram-negative opportunistic pathogen, *Pseudomonas aeruginosa*, is a typical biofilm-forming microbe, and this characteristic allows it to thrive in a diverse range of natural and nosocomial niches (Driscoll et al., 2007). *P*. *aeruginosa* is a common cause of severe infections in wounds, eyes and lungs, and it is often difficult to treat, due to the high prevalence of multi-drug resistance (Breidenstein et al., 2011). The quorum sensing (QS) system is a population density-dependent regulatory system that enables cell-to-cell communication and coordinated control of gene expression (Fuqua et al., 1994). Co-ordination of gene expression through QS is an important determinant of virulence and drug-resistance in *P. aeruginosa* (Van Delden and Iglewski, 1998). It is proposed that inhibitors of the QS system may act as effective anti-microbial agents by deregulating these determinants of pathogenesis and thus increasing the effectiveness of host defenses and antibiotic treatment (Fothergill et al., 2012).

*P. aeruginosa* utilizes two interconnected QS systems, termed Las and Rhl (Schuster et al., 2013), which are regulated by *N*-acyl-homoserine lactones (AHLs, also termed *P. aeruginosa* autoinducers; PAIs). The *las* system is the predominant of the two QS systems and consists of the LasI and LasR proteins (Gambello and Iglewski, 1991). LasI synthesizes the AHL molecule, *N-3-oxododecanoyl-L-homoserine* lactone (3OC12-HSL, PAI-1), which is bound by the transcription regulator, LasR (Pearson et al., 1994). LasR directly regulates the expression of up to 74 genes, including *lasI* (Gilbert et al., 2009). In the analogous Rhl system, RhlI synthesizes *N*-butyryl-L-homoserine lactone (C4-HSL, PAI-2) and RhlR acts as the sensor/transcriptional regulator (Brint and Ohman, 1995).

The concentration of the population, and thus the two concentration of the two AHL molecules, determines the expression of multiple proteins related to virulence, drug resistance, motility and biofilm development (Williams and Camara, 2009).

Both natural and synthetic molecules that block QS have been shown to inhibit effectively QS systems, both *in vitro* (Pejin et al., 2014) and *in vivo* (Wu et al., 2004). For example, it was previously demonstrated that a sub-MIC level of the antibiotic azithromycin (AZM), which was sufficient to inhibit QS, was also effective in treating *P. aeruginosa* infections (Imperi et al., 2014).

*Houttuynia cordata* Thunb (Saururaceae family) is an edible plant used in traditional Chinese medicine for the treatment of a wide range of infectious diseases, including pneumonia (Gao et al., 2009, 2010; Li et al., 2014). The major constituent of the volatile oil derived from *H. cordata*, sodium houttuyfonate [SH, CH_3_(CH_2_)_8_COCH_2_CHOHSO_3_Na] is a product of the addition reaction of sodium bisulfite and houttuynin [i.e., decanoyl acetaldehyde, CH_3_(CH_2_)_8_COCH_2_CHO] (Wang et al., 2002). SH is the active compound of the *Houttuynia* plant, the healing properties of which have been recorded in ancient Chinese medical writings (Gao et al., 2010). SH is mainly used for treating purulent skin infections, respiratory tract infections, including pneumonia in elderly patients, and chronic bronchitis (Wang et al., 2002). However, despite its widespread and effective use, the mode of action remains unknown. In previous studies we reported that SH can inhibit biofilm formation and motility of *P. aeruginosa* (Shao et al., 2012, 2013a,b). We found that SH can effectively prevent biofilm formation of *P. aeruginosa, Staphylococcus epidermidis* and *Candida albicans* (Shao et al., 2013a,b) and acts synergistically with the broad-spectrum antibiotic, levofloxacin (Shao et al., 2012). The mode of action of SH, however, remains unknown. Therefore, in this study we focused on the effect of SH on quorum sensing. Here, we investigate the putative role of SH as a QS-inhibitor in *P. aeruginosa*.

## Materials and methods

### Bacterial strains, media and growth conditions

*P. aeruginosa* strain ATCC 27853, obtained from the National Institute for the Control of Pharmaceutical and Biological Products (NICPBP, Beijing, China) was inoculated in Luria–Bertani (LB) broth (Aoboxing Bio-tech, Beijing, China) and grown under standard conditions (37°C, 220 rpm). *Chromobacterium violaceum* strain CV026 (McClean et al., 1997) was grown in LB broth supplemented with 1% agar, fetal bovine serum (20%, w/v), L-tryptophan (0.007%, w/v) and/or kanamycin (20 μg/ml) as appropriate which is modified from original medium of McClean et al. (1997). In the modified medium, fetal bovine serum and L-tryptophan are added to the original medium, because L-tryptophan is known to increase the purple pigment production of *C. violaceum* (Demoss and Evans, 1959) and fetal bovine serum was observed by us to accelerate the growth speed of *C. violaceum* (data not shown).

For measurement by spectrophotometry, cells were harvested at 24 h after inoculation by centrifugation at 4600 × g for 10 min. The supernatant was discarded and the pellet was resuspended in sterile saline solution for optic density detection at 600 nm (OD_600_) in a UV spectrophotometer. The absorbance of cell suspensions was adjusted to 0.05 for further experiments.

### MIC determination

A micro-broth dilution method (Wiegand et al., 2008) was adopted to test the minimum inhibitory concentrations (MICs) of SH and AZM. The assay was performed using 96-well plates and consisted of a gradient of inhibitor concentrations, i.e., 2048, 1024, 512, 256, 128, 64, 32, 16, 8, 4, 2, 1, 0.5, and 0.25 μg/ml, of equal final volume (100 μl), and 100 μl of bacterial suspension (final concentration 0.75 × 10^6^ CFU/ml). After mixing, the plates were cultured for 24 h at 37°C and the OD_600_ was measured. Each assay was performed in triplicate.

### Growth inhibition assay

Antibiotics were added to a *P. aeruginosa* suspension (1 × 10^6^ CFU/ml) in a constant-temperature shaker (37°C) at 220 rpm. Drugs were added into the medium at the following concentrations: 64, 128, 256, and 512 μg/ml (1/8–1 × MIC) and 64 μg/ml (1 × MIC) AZM. Growth inhibition was measured using OD_600_ relative to the control culture (no drug) over a 72 h period and it was further quantified by plating the cultures and counting the CFU at 24 h and 72 h.

### Extraction of signaling molecules

*P. aeruginosa* was grown in 100ml of medium under standard conditions for 72 h, followed by centrifugation (10,800 × g, 10 min, 4°C) and subsequent transfer of the cleared supernatant into a clean flask. An equal volume of ethyl acetate was added, with mixing, and the organic phase was separated by centrifugation (10,800 × g for 15 min at 4°C). The organic phase was transferred into another clean flask and initially concentrated by evaporation to a 1 ml volume through heating in a water bath at 37°C. The remaining solution was further concentrated and stored in a sterile EP tube at −80°C. Each concentration was repeated in triplicate.

### Biological detection of signaling molecules

Detection of AHLs was determined on agar plates employing the biosensor strain *C. violaceum* CV026, which produces a purple pigment only in response to exogenously added AHLs (McClean et al., 1997). Overnight cultures (LB broth supplemented with 20 μg/ml kanamycin, 0.007% (w/v) L-tryptophan and 20% (w/v) fetal bovine serum) were mixed (20% (v/v)) with LB broth containing 2% agar and poured into plates. Once set, a 20 μl drop of signaling molecule solution was added in the center of the plates and the plates were incubated at 48°C for 24 h for development and observation of the violet color zone. Liquid cultures containing the signaling molecule solution were harvested after 24 h growth (220 rpm, 37°C). A 300 μl sample of the culture was transferred to a centrifuge tube and 300 μl of 10% (w/v) SDS was added. The cells were vortex-mixed for 5 s and then 2.1 ml of 98% ethanol was added. The supernatant was harvested (10,800 × g, 10 min, 4°C) and the OD_580_ was measured. Each experiment was repeated in triplicate.

### Gene expression analysis

Approximately 0.75 × 10^6^ CFU (in a 200 μl volume) were used to inoculate 5 ml broths containing a range of SH concentrations, alone or in combination with AZM. Cultures were grown for 72 h, the cells were harvested by centrifugation (10,800 × g, 1 min) and the supernatant was discarded. Total RNA was extracted using an RNAprep Pure Cell/Bacteria Kit (Code No. DP430, TIANGEN, China), according to the manufacturer's guidelines. A FastQuant RT Kit (Code No. KR106, TIANGEN, China) was used to remove genomic DNA, and the purified RNA (2 μg) was used for reverse-transcription. The oligonucleotide primers used are designed and listed in Table 1. Reverse-transcription polymerase chain reaction (RT-PCR) was performed using LA Taq (Takara, Japan). The conditions were as follows: one step of 5 min at 95°C and 35 cycles of 95°C for 30 s, 56°C for 30 s and 72°C for 30 s. The resulting cDNA was electrophoresed on 1% agarose gel and then imaged. Quantitative RT-PCR (qRT-PCR) was performed using Realtime PCR Master Mix (SYBR Green) (Code No. QPK-201, QIAGEN, Germany) using the following conditions: one step of 60 s at 95°C and 40 cycles of 95°C for 15 s, 56°C for 15 s and 72°C for 30 s. The calculated cycle threshold (C_T_) of each gene was normalized to the C_T_ for *rpoD* amplified from the corresponding sample. The reactions for RT-PCR and qRT-PCR were performed in ABI 9700 and ABI PRISM thermal cyclers, respectively (Applied Biosystems, USA). Fold-changes in gene expression were calculated according to the 2^−ΔΔCT^ method (Livak and Schmittgen, 2001).

**Table 1:** **Oligonucleotide primers used during RT-PCR**.

**Gene**	**Forward (5′–3′)**	**Reverse (5′–3′)**
*lasI*	TTGCTCGCCGCACATC	GGCACGGATCATCATCTT
*rhlI*	ATCCGCAAACCCGCTAC	GCAGGCTGGACCAGAATAT
*lasR*	CATCGTCGGCAACTACCC	GCGCACCACTGCAACACT
*phzM*	GACATGGTGCTGTTCTACGG	TGGAATGCCAGGTTGCTC
*lasA*	CTACAGCATCAACCCGAAAG	TAGCGCCGCGACAACT
*pslA*	TACCGGGCCTGGATGA	CGGCAGCGAGTTGTAGTT
*lasB*	GTTCTATCCGCTGGTGTCG	CGCTGCCCTTCTTGATG
*rsmA*	AGACCCTGATGGTAGGTG	AATGGTTTGGCTCTTGAT
*gacA*	AACTGGCCCGCGAACT	GCGCCCTTGGTCATGTAG
*mexA*	TCCCTGAAGCTGGAGGACG	TGCTGCGGAGCGAGGAT
*ropD*	AGGCCGTGAGCAGGGAT	GGTGGTGCGACCGATGT

### Pyocyanin quantification assay

After 24 h or 72 h cultivation, the culture of *P. aeruginosa* was centrifuged at 10,800 × g for 1 min. The resulting supernatant (5 ml) was mixed with chloroform (3 ml) and then centrifuged at 4600 × g for 10 min. The chloroform phase was transferred to another centrifuge tube, mixed with 1 ml 0.2 M HCl and then centrifuged at 4600 × g for 10 min. The upper phase was taken to detect OD_520._ The A OD_520_ reading was normalized by dividing by the final OD_600_ reading of the culture. The quantity of pyocyanin was calculated by multiplying OD_520_ by 17.072 (Kong et al., 2005).

### LasA staphylolytic assay

LasA protease activities of different groups were measured by measuring the ability of stationary-phase *P. aeruginosa* culture supernatant to lyse boiled *Staphylococcus aureus* (Kessler et al., 1993). The LasA staphylolytic assay was performed according to Kong et al. (2005).

### Statistical analysis

All data were analyzed by SPSS 17.0 statistical software, and expressed as mean ± standard deviation (SD). Different group of data were compared by Student's *T*-test. All experiments were carried out at least in triplicate.

## Results

### Effect of SH on growth of *P. aeruginosa*

**Figure 1:**
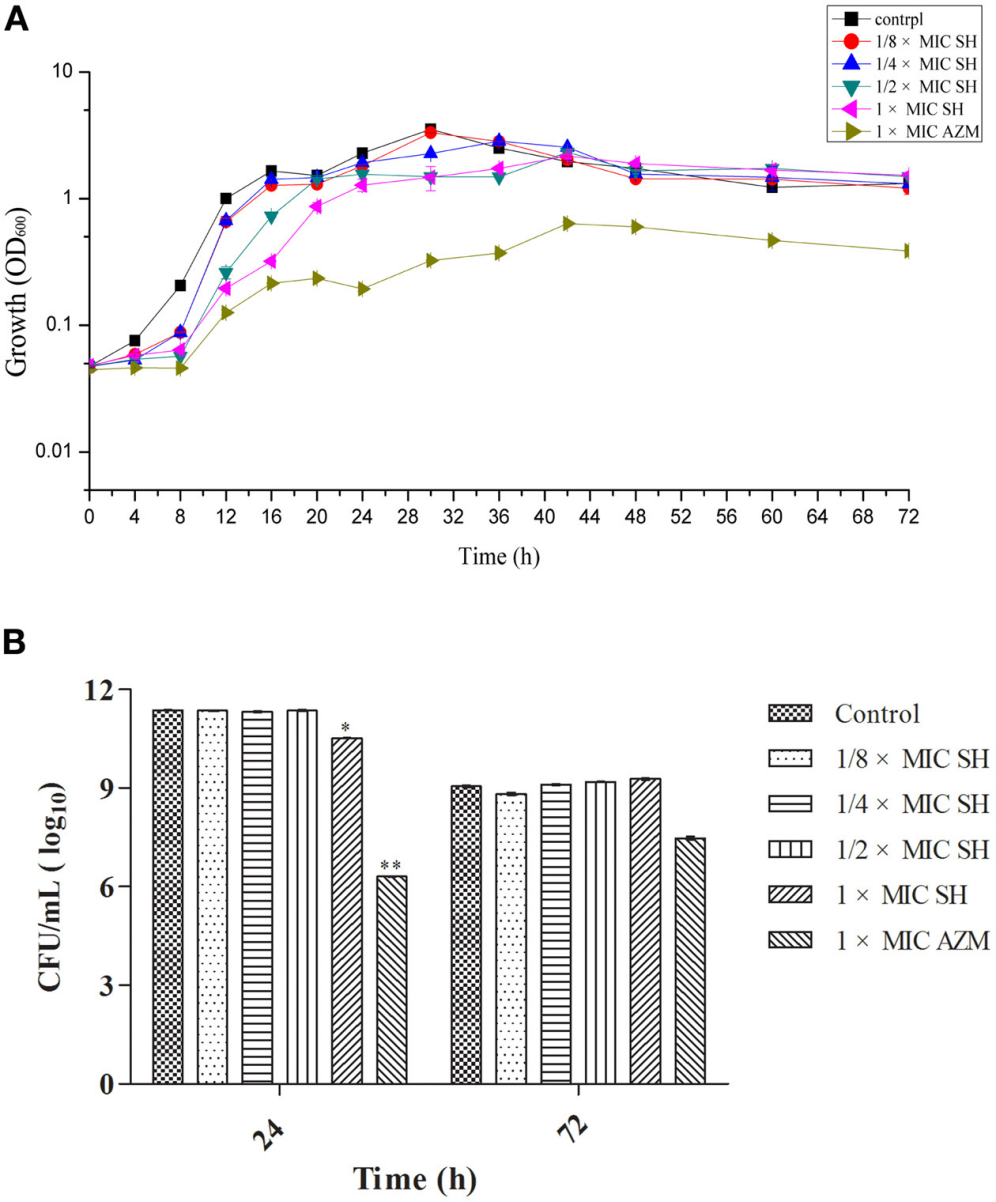
**Effect of SH on growth of *P. aeruginosa*. (A)** Growth curves of *P. aeruginosa* treated with SH varying concentrations of SH and AZM (512 μg/ml (1 × MIC) SH, 256 μg/ml (1/2 × MIC) SH, 128 μg/ml (1/4 × MIC) SH, 64 μg/ml (1/8 × MIC), and 64 μg/ml (1 × MIC) AZM). The control contained no SH and AZM. OD_600_ numbers of growth is calculated by “ln” to draw the presented semi-log plot. **(B)** CFU of *P. aeruginosa* treated with SH for 24 h and 72 h. The drug concentration of treatments was same as **(A)**. The ^*^ and ^**^ indicate the *p*-value < 0.05 and 0.01, respectively.

We first determined the MIC for SH and the broad-spectrum antibiotic AZM, which is known to inhibit QS at sub-MIC concentrations (Bala et al., 2011) using the Micro-broth dilution method. We found the MICs for *P. aeruginosa* strain ATCC27853 to be 512 μg/ml and 64 μg/ml, respectively. The high MIC for SH would limit the clinical use of SH as a growth inhibitor to treat *P. aeruginosa* infections. The growth curves of *P. aeruginosa* treated by SH (Figure 1A) showed that SH inhibits the growth of *P. aeruginosa* in the early growth stages, before 30 h, at all tested concentrations. After 30 h, the solutions containing 64 μg/ml and 128 μg/ml SH showed no inhibitory activity toward *P. aeruginosa* and only those solutions containing >256 μg/ml showed inhibitory activity. In the declining stage of the growth curve, the concentration of *P. aeruginosa* was independent of SH, with no significant difference between SH-containing cultures and the control. Measurement of CFU at 24 and 72 h showed that only cultures containing the full SH MIC (512 μg/ml) demonstrated lower CFU at 24 h than the control group, while at 72 h no differences were detected (Figure 1B). As expected, the growth curve and CFU results of cultures containing the AZM showed significant growth differences when compared with the control group. These results indicate that SH alone is inefficient at inhibiting growth under the conditions tested. We therefore assessed the specific effect of SH on QS.

### Effect of SH on QS-regulated systems

**Figure 2:**
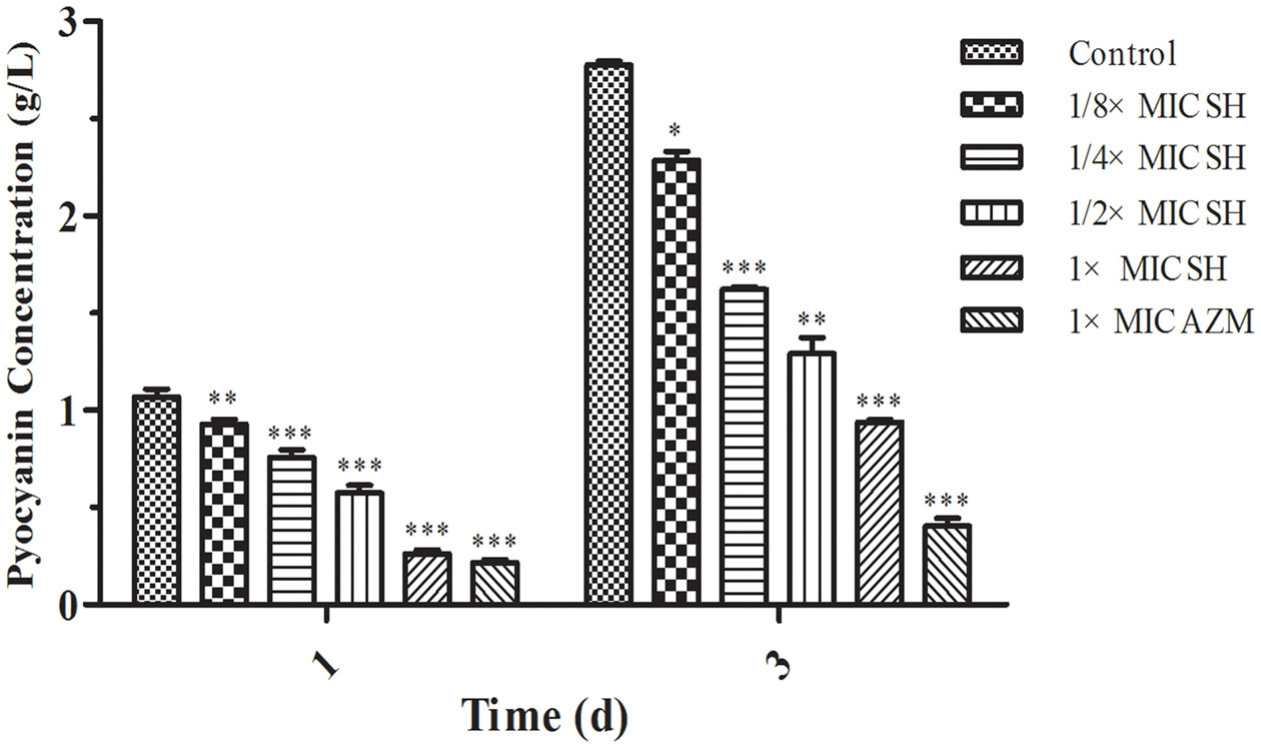
**Pyocyanin production of *P. aeruginosa* in response to SH**. Pyocyanin production of *P. aeruginosa* cultured under different drug concentrations was measured at 1 d and 3 d. The drug concentration of treatments was as follows: Control (without any drugs), 512 μg/ml (1 × MIC) SH, 256 μg/ml (1/2 × MIC) SH, 128 μg/ml (1/4 × MIC) SH, 64 μg/ml (1/8 × MIC), and 64 μg/ml (1 × MIC) AZM. The statistical significances of all data were reported to be compared with the control group. The ^*^, ^**^, and ^***^ indicate the *p*-value < 0.05, 0.01, and 0.001, respectively.

Addition of SH was shown to affect production of the QS-regulated chromogenic toxin, pyocyanin, the presence of which is indicated by the green coloration of the growth medium (Figure 2). We observed that addition of SH reduced the green pigmentation of the medium to a level similar to that of the positive control, AZM (Figure S1). Purification and quantification of the pyocyanin in the culture supernatants again demonstrated that addition of SH dose-dependently inhibits pyocyanin production (Figure 2), which in *P. aeruginosa* is regulated by the Las system (Williams and Camara, 2009).

To further investigate the potential for SH to disrupt QS, we used the Gram-negative bacterium *C. violaceum*, which produces a water-soluble purple dye (violacein) under the control of an AHL-controlled QS system (McClean et al., 1997). Specifically, we used a mutant derivative *C. violaceum* CV026 that lacks the gene (*cviI*) required to produce AHL, thus producing violacein only in response to exogenously supplied AHLs (McClean et al., 1997) and providing a convenient tool with which to screen QS inhibitors (Blosser and Gray, 2000).

**Figure 3:**
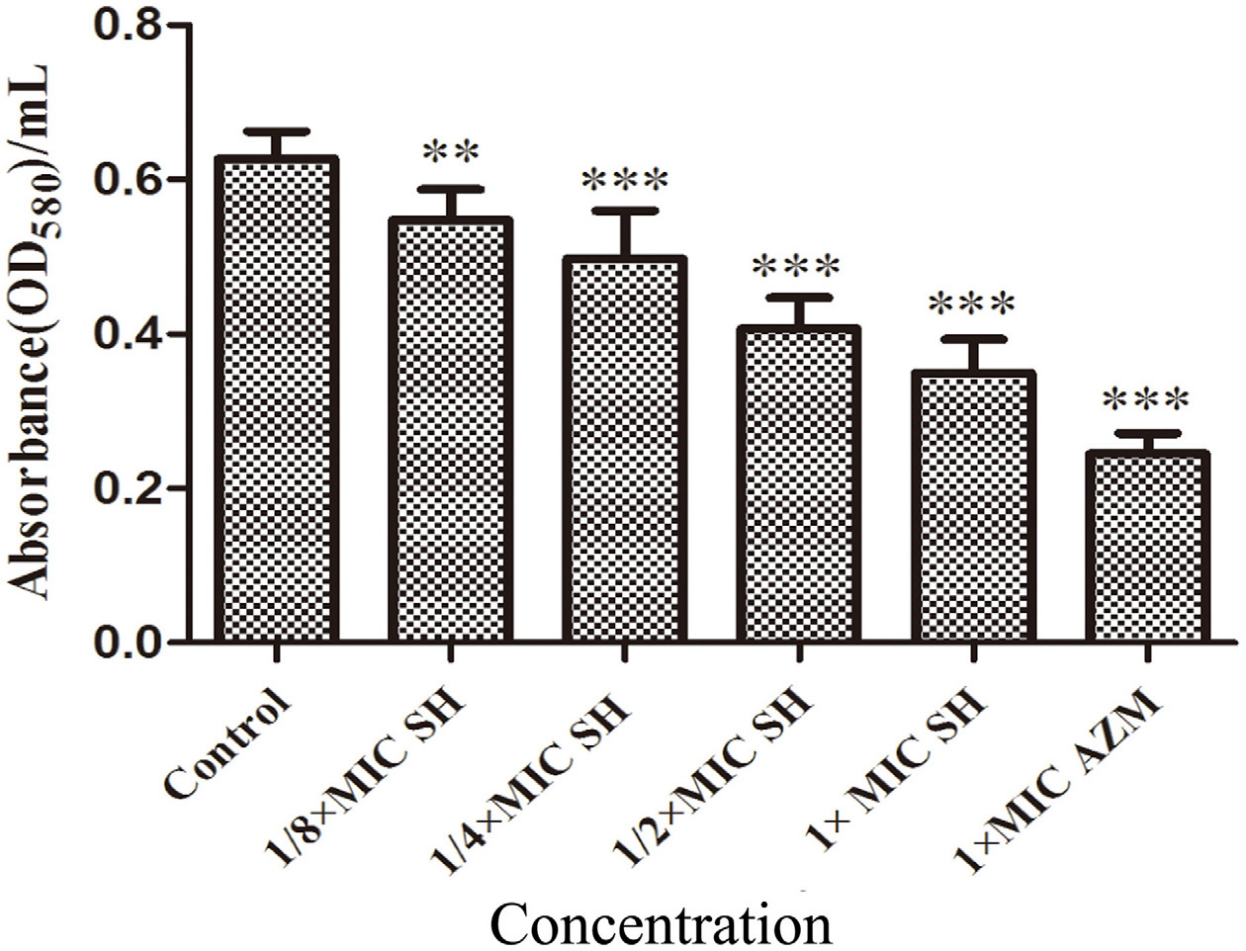
**Effect of SH on the QS-regulated production of violacein**. OD value of violet pigment treated by SH. The drug concentration of treatments was as follows: Control (without any drugs), 512 μg/ml (1 × MIC) SH, 256 μg/ml (1/2 × MIC) SH, 128 μg/ml (1/4 × MIC) SH, 64 μg/ml (1/8 × MIC), and 64 μg/ml (1 × MIC) AZM. The ^**^ and ^***^ indicate the *p*-value < 0.01 and 0.001, respectively.

SH had a clear dose-dependent inhibitory effect on the production of violacein, indicating that SH was capable of blocking QS regulation in the system (Figure S2), which we further corroborated by spectrophotometric analysis after growth in liquid culture (Figure 3).

### Expression of the AHL biosynthesis genes in response to SH

**Figure 4:**
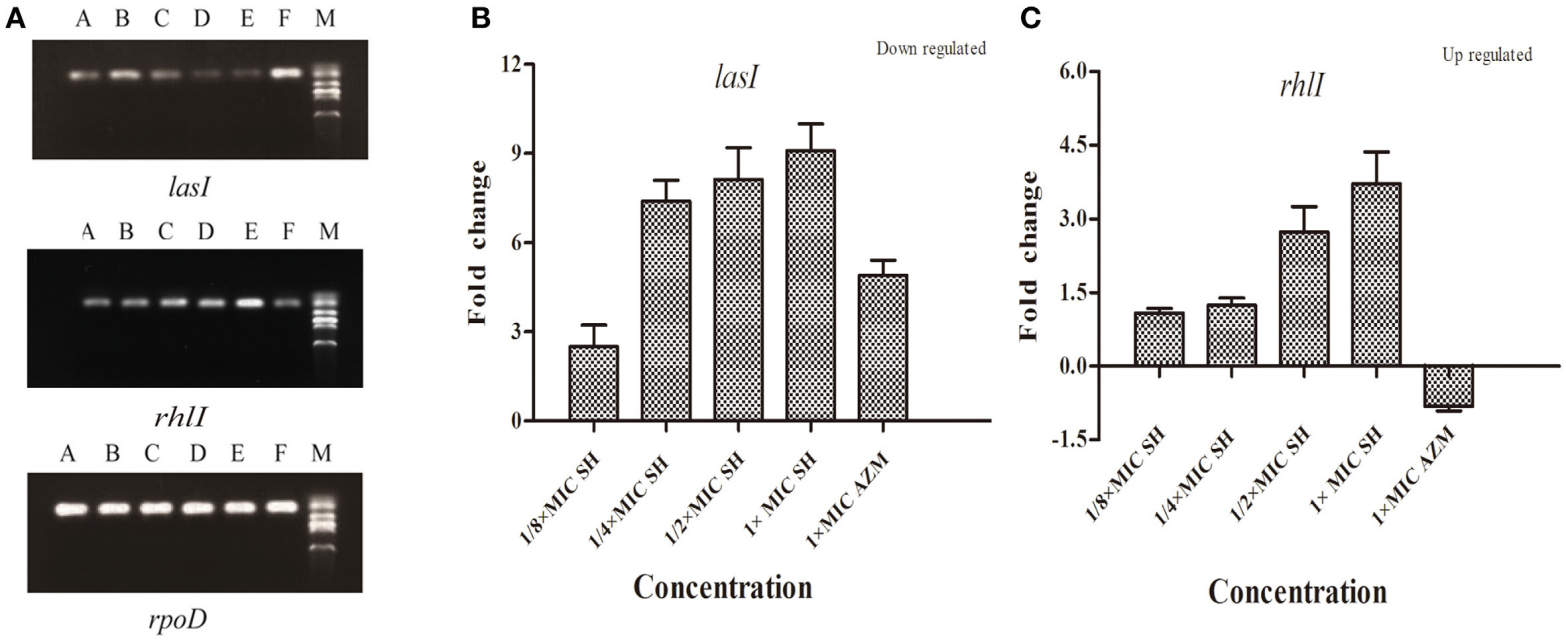
**Effect of SH on the expression of *lasI* and *rhlI*. (A)** RT-PCR of the *lasI*,*rhlI* and *rpoD* genes. The lanes from right to left were as follows: Marker, Control, 512 μg/ml (1 × MIC) SH, 256 μg/ml (1/2 × MIC) SH, 128 μg/ml (1/4 × MIC) SH, 64 μg/ml (1/8 × MIC), and 64 μg/ml (1 × MIC) AZM. **(B)** Quantitative RT-PCR of the *lasI*. **(C)** Quantitative RT-PCR of the *rhlI*. Expression of the house-keeping gene, *rpoD*, was used as the internal control for each sample. The drug concentration of treatments was as follows: 512 μg/ml (1 × MIC) SH, 256 μg/ml (1/2 × MIC) SH, 128 μg/ml (1/4 × MIC) SH, 64 μg/ml (1/8 × MIC), and 64 μg/ml (1 × MIC) AZM.

To investigate the effects of SH on the expression AHL biosynthesis genes *lasI* and *rhlI*, we performed RT-PCR and qRT-PCR experiments. The RT-PCR results (Figure 4A) showed that *lasI* expression was down-regulated by the presence of SH. The qRT-PCR results revealed a dose-dependent down-regulation of *lasI* in response to increasing SH concentrations (Figure 4B), with a fold change in *lasI* levels of 2.50, 7.40, 8.13, 9.09 in response to the presence of 64, 128, 256, and 512 μg/ml SH, respectively. AZM, which was previously shown to effectively inhibit QS, down-regulated *lasI* 4.91 fold at its MIC of 64 μg/ml. Unexpectedly, we found that the expression *rhlI* was up-regulated by SH (Figure 4C) indicating that SH has a specific, dose-dependent effect on expression of the main AHL biosynthesis gene, *lasI*.

### Expression of LasR and related genes and in response to SH

**Figure 5:**
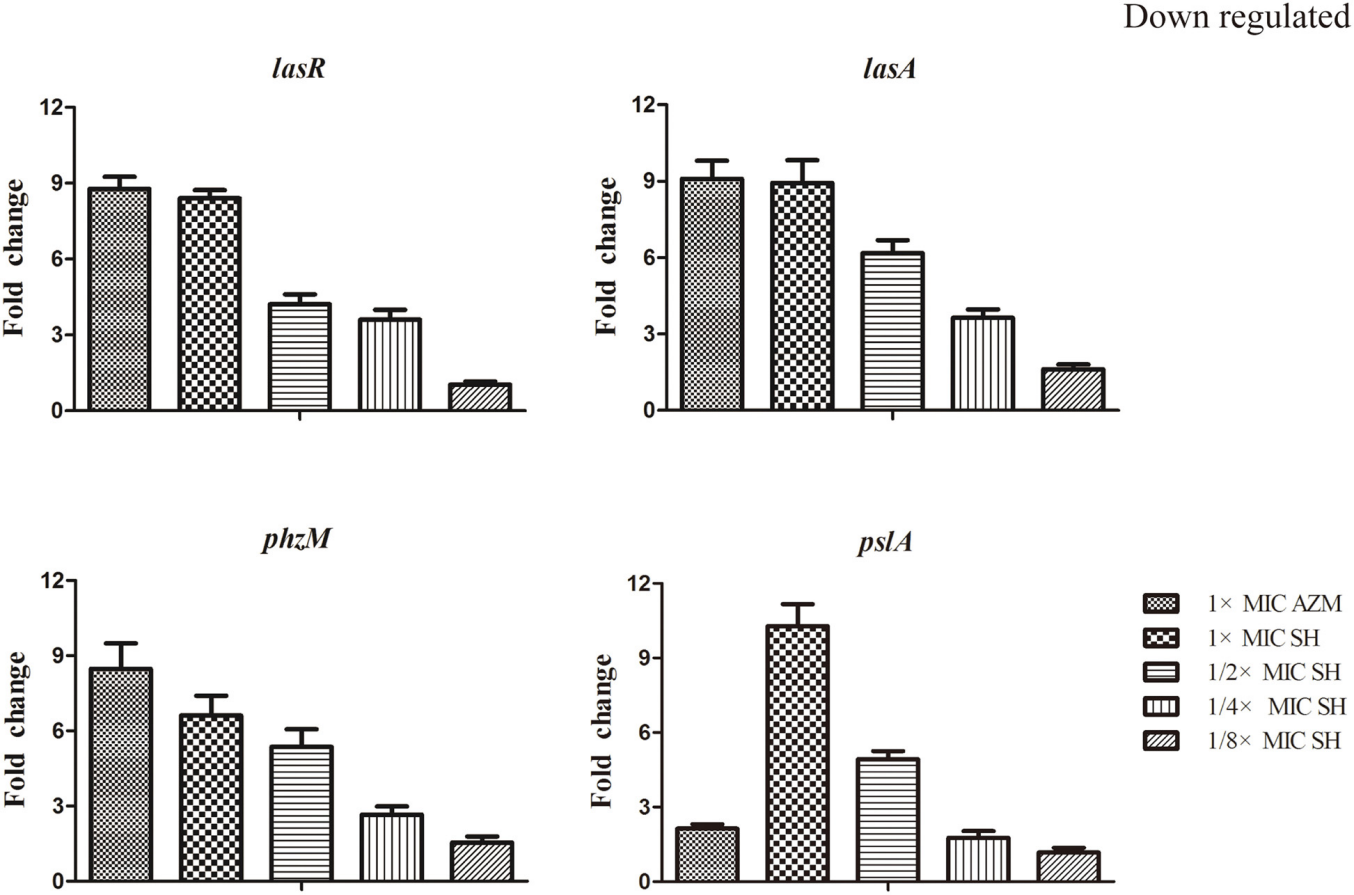
**Repression of SH on the expression of LasR and genes regulated by Las system**. The expression of *lasR, lasA, phzM* and *pslA* were monitored in response to SH treatment. Expression of the house-keeping gene, *rpoD*, was used as the internal control for each sample. The drug concentration of treatments was as follows: 512 μg/ml (1 × MIC) SH, 256 μg/ml (1/2 × MIC) SH, 128 μg/ml (1/4 × MIC) SH, 64 μg/ml (1/8 × MIC), and 64 μg/ml (1 × MIC) AZM.

LasR is the key regulator factor of the Las system of *P. aeruginosa* (Williams and Camara, 2009). The qRT-PCR results showed that the expression of *lasR* gene was strongly down-regulated by SH treatments (Figure 5). Furthermore, we found that *lasA* and *pslA*, which are regulated by LasR, were down-regulated in response to SH in a concentration-dependent manner (Figure 5). And the pyocyanin biosynthesis gene, phzM was also down-regulated by Sh in the dose-dependent manner (Figure 5). These results indicate that SH causes inhibition of many QS-regulated genes, including the main QS regulator, *lasR*. In addition, we also detected the expression of *lasB, gacA, rsmA*s, and *mexA* related in virulence factor, virulence regulation and drug resistance under the SH treatment. However, expression of these four genes is not significantly affected by SH (Figure S3).

**Figure 6:**
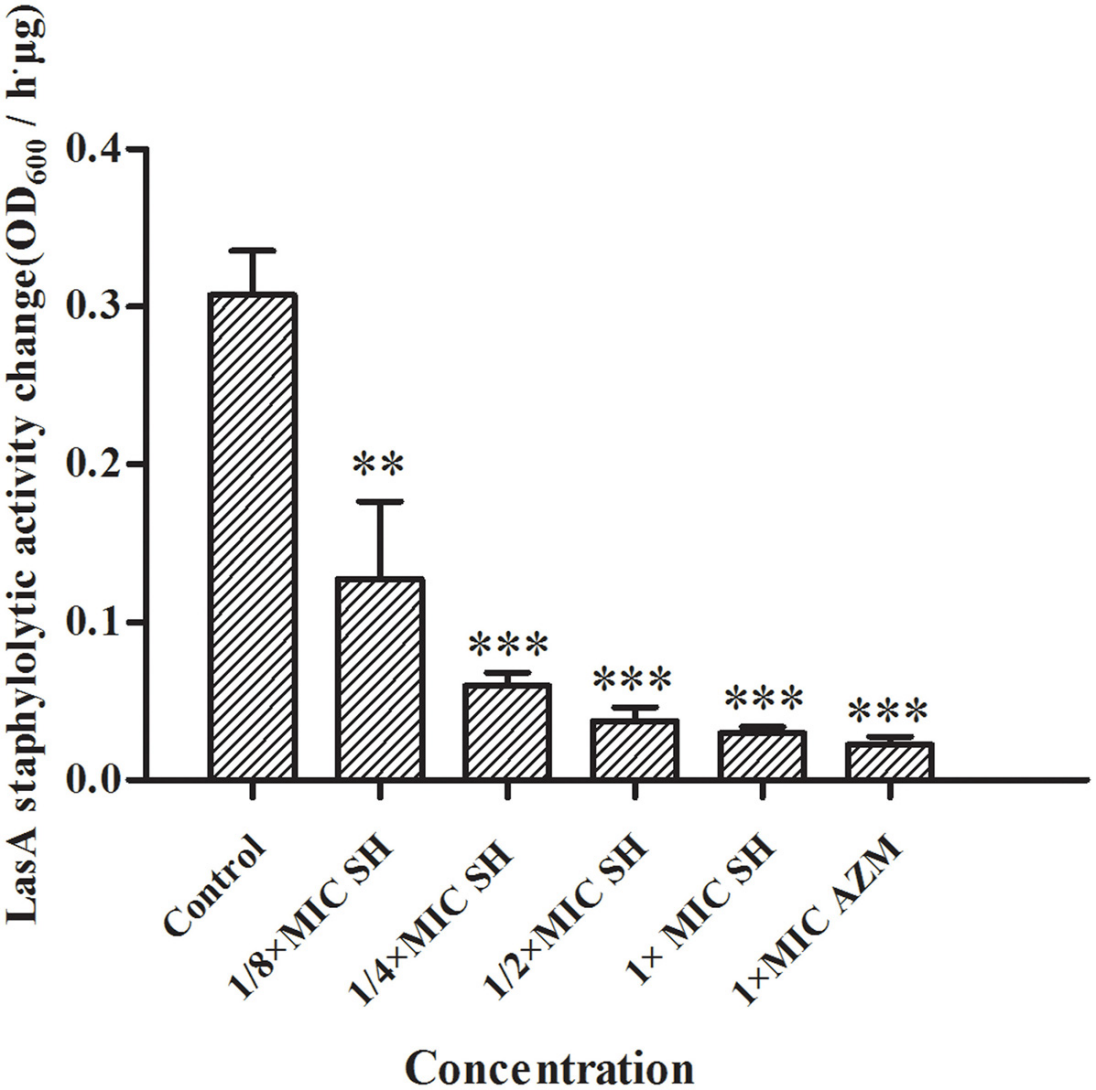
**Inhibition of SH against LasA activity**. The drug concentration of treatments were as follows: Control (without any drugs), 512 μg/ml (1 × MIC) SH, 256 μg/ml (1/2 × MIC) SH, 128 μg/ml (1/4 × MIC) SH, 64 μg/ml (1/8 × MIC), and 64 μg/ml (1 × MIC) AZM. The ^**^ and ^***^ indicate the *p*-value < 0.01 and 0.001, respectively.

LasA is an important virulence factor of *P. aeruginosa* and is positively regulated by LasR. Considering our observation of significant down-regulation of *lasR* in response to SH, we decided to monitor the effect of SH on production of LasA. We found that LasA enzymatic activity was significantly repressed by SH, even at concentrations below those that inhibit growth (Figure 6). Considering that SH also inhibited production of the toxin and important virulence factor, pyocyanin (Figure 2), our data suggest SH can be used to significantly inhibit the production of key *P. aeruginosa* virulence factors, independent of a direct effect on growth rate.

## Discussion

*P. aeruginosa* is one of the ESKAPE pathogens (*Enterococcus faecium, Staphylococcus aureus, Klebsiella pneumoniae, Acinetobacter baumanii, P. aeruginosa, and Enterobacteriaceae*) emphasizing their strong capacity to “escape” from routine antibacterial treatments (Boucher et al., 2009). The quorum sensing system is a key regulatory system which is responsible for the multi-drug resistance and pathogenesis of *P. aeruginosa* (Van Delden and Iglewski, 1998). Thus, development of quorum sensing inhibiting agents is one of the key areas in the Pseudomonas research field (Fothergill et al., 2012).

Here we have demonstrated that SH can effectively inhibit the production of the QS-regulated virulence factors, LasA and pyocyanin, with sub-MIC concentrations sufficient to inhibit QS-regulated systems independent of an effect on growth. We found that SH causes a specific, dose-dependent repression of two components of the Las QS system, the AHL biosynthesis gene, *lasI*, and the transcriptional regulator of QS LuxR-type receptor, *lasR*. In line with these results, we also found that SH repressed expression of the *lasR* regulated genes *lasA*, and *pslA* (Gilbert et al., 2009; Jimenez et al., 2012). Among them, *lasA* encode secreted protease LasA which is a virulence factor of *P. aeruginosa* (Kessler et al., 1993), and *pslA* is the first gene of *psl* operon encoding the biosynthesis enzyme of Psl polysaccharide of biofilm matrix (Colvin et al., 2012). *PhzM* is responsible for pyocyanin production (Huang et al., 2009) *LasA* and *pslA* are directly and positively regulated by LasR in *P. aeruginosa*, and pyocyanin production is positively regulated by LasR (Gilbert et al., 2009; Jimenez et al., 2012). However, *lasB, gacA, rsmA* and *mexA* were not found to be down-regulated by SH treatment. *LasB* codes for the elastase, LasB which plays a role in pathogenesis of *P. aeruginosa* respiratory infections by rupturing the respiratory epithelium (Azghani, 1996) and is regulated by LasR and RhlR in combination (Gilbert et al., 2009; Jimenez et al., 2012). *GacA* and *rsmA* enconde regulators of GacA and RsmA of Gac/Rsm signal transduction pathway which positively controls quorum sensing in *P. aeruginosa* (Heeb et al., 2002). GacA is the positive regulator of *lasR* and *rhlR*, and RsmA negatively controls the *lasI* and *rhlI* (Williams and Camara, 2009). *MexA* is the first gene of operon enconding MexAB-OprM efflux pump responsible for intrinsic drug resistance of *P. aeruginosa* (Breidenstein et al., 2011; Poole, 2013). Expression of *mexAB-oprM* is positively regulated by C4-HSL in Rhl system (Evans et al., 1998; Sawada et al., 2004; Sugimura et al., 2008), and MexAB-OprM regulates QS in *P. aeruginosa* by controlling accessibility of non-cognate acyl-HSLs to LasR (Minagawa et al., 2012). Thus, *lasB, gacA, rsmA*, and *mexA* are not regulated by Las system solely or directly, and not down-regulated by SH treatment. Taken together, our results imply that SH can specifically inhibits the Las system and related genes expression.

**Figure 7:**
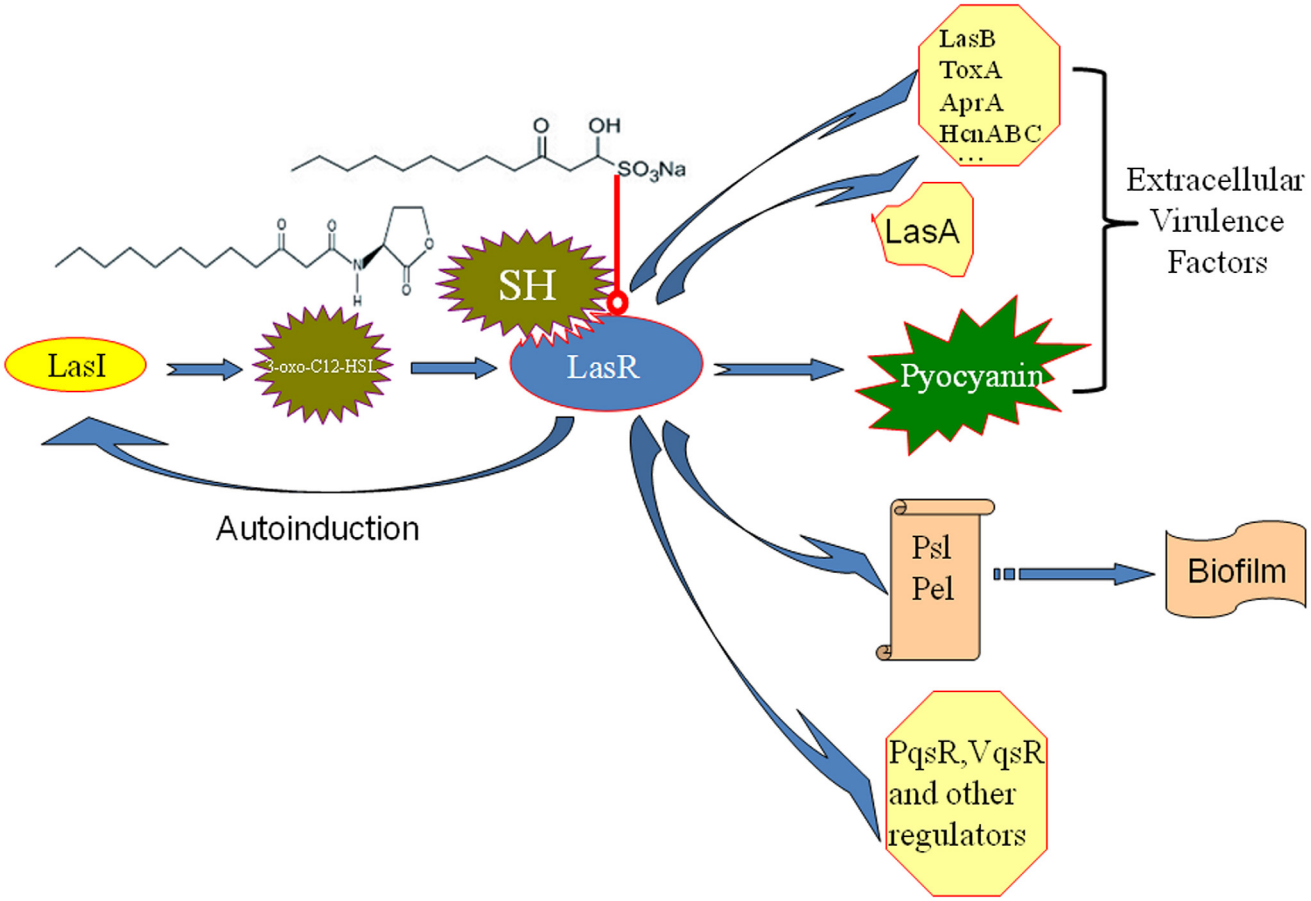
**Putative mechanism for the inhibition of *P. aeruginosa* QS by SH**. The similarity in the chemical structures of SH and 3-oxo-C12-HSL suggests that SH may compete with for signal molecule binding of LasR and thus specifically inhibit the sensing of this molecule to reduce the enzyme activity of LasR. Then, LasA and LasI activity, pyocyanin production and biosynthesis of biofilm matrix component Psl, which are positively regulated by LasR, are inhibited by SH treatment. Therefore, pathogenicity of *P. aeruginosa* may be attenuated by repression of extracellular virulence factors and biofilm formation of SH. LasR also positively regulates other extracellular virulence factors (LasB, ToxA, AprA, HcnABC), biofilm matrix component Pel, regulators (PqsR, VqsR, and so on) alone or combined with RhlR (Williams and Camara, 2009). Las system has a positive feedback (autoinduction) that at high population densities by increasing in QS signal molecule production and consequently enhanced expression of the target genes (Williams and Camara, 2009). Blue arrow besides LasR stands for the positive regulatory effect of LasR. The arrow between LasI and 3-oxo-C12-HSL means that LasI is responsible for biosynthesis of 3-oxo-C12-HSL. The arrow between 3-oxo-C12-HSL and LasR means that 3-oxo-C12-HSL bind of LasR to induce the enzyme activity of LasR. The arrow between polysaccharides, Psl, Pel and biofim stands for that Psl and Pel is the main matrix components of biofilm in *P. aeruginosa*. Red line with a round at the end stands for inhibiting effects.

Molecules modulating QS LuxR-type receptors to interfere with bacterial virulence and biofilms is the most intensively investigated in the antiquorum sensing research (Wang and Ma, 2013). AHL Analogs (Smith et al., 2003), furanones (Givskov et al., 1996), benzoheterocyclics (Peters et al., 2003), 4-Nitropyridine-N-oxide (Rasmussen et al., 2005), thimerosal and phenyl percuric nitrate (Taha et al., 2006), azithromycin (Imperi et al., 2014), ceftazidime and ciprofloxacin (Skindersoe et al., 2008), tobramycin (Garske et al., 2004), solenopsin A (Park et al., 2008), and andrographolides (Ma et al., 2012) were found to possess the antiquorum sensing activity by modulate QS LuxR-type receptor, i.e., LasR or RhlR. Rather than affecting both the Las and Rhl QS systems in the same manner, our data also revealed the up-regulation of the Rhl system regulator, *rhlR*, indicating a possible compensation of the Las QS system by the Rhl system (Figure 4). In the available antiquorum sensing drugs, the chemical structure of synthetic AHL analogs is also close to SH. Among them, the ribolactam analogs and cyclic azahemiacetals were found to significantly block Las system at all concentrations tested and to moderately stimulate rhl, which is also similar to SH (Malladi et al., 2011). Thus, the results suggest that SH is a natural AHL analog. Interestingly, two other AHL analog compounds with 12-carbon alkyl tails have also been identified as specific inhibitors of the Las system (Muh et al., 2006), while AZM and 14-alpha-lipoyl andrographolide were shown to inhibit both the Las and Rhl systems (Ma et al., 2012; Imperi et al., 2014). While AZM is believed to affect QS through a more general effect on translation (Imperi et al., 2014), the similarity in the chemical structures of SH and AHL suggests that SH may compete with 3-oxo-C12-HSL for binding of LasR and thus specifically inhibit the sensing of this molecule (Figure 7).

In conclusion, our results demonstrate that the natural products of plants, especially those used in traditional medicine, could be an important source of clinically-relevant quorum sensing inhibitors.

### Conflict of interest statement

The authors declare that the research was conducted in the absence of any commercial or financial relationships that could be construed as a potential conflict of interest.

## Supplementary material

Figure S1 **Color change of *P. aeruginosa* in response to SH**. The color change of *P. aeruginosa* in liquid culture with various concentrations of SH [Control (without any drugs) (a), 64 μg/ml (1/8 × MIC) SH (b), 128 μg/ml (1/4 × MIC) SH (c), 256 μg/ml (1/2 × MIC) SH (d), 512 μg/ml (1 × MIC) SH (e) and 64 μg/ml (1 × MIC) AZM (f)].

Figure S2 **Plate assay demonstrating SH affecting AHL production**. The AHLs from different groups of *P. aeruginosa* of Control (a), 64 μg/ml (1/8 × MIC) SH (b), 128 μg/ml (1/4 × MIC) SH (c), 256 μg/ml (1/2 × MIC) SH (d), 512 μg/ml (1 × MIC) SH (e) and 64 μg/ml (1 × MIC) AZM (f) were extracted and added into the center of CV026 plates.

Figure S3 **Effect of SH on the expression of *lasB, gacA, rsmA* and *mexA***. The expression of *lasB, gacA, rsmA*, and *mexA* were monitored in response to SH treatment. Expression of the house-keeping gene, *rpoD*, was used as the internal control for each sample. The drug concentration of treatments was as follows: 512 μg/ml (1 × MIC) SH, 256 μg/ml (1/2 × MIC) SH, 128 μg/ml (1/4 × MIC) SH, 64 μg/ml (1/8 × MIC) and 64 μg/ml (1 × MIC) AZM.
