# Supplementary figures and images for: Sodium houttuyfonate affects production of N-acyl homoserine lactone and quorum sensing-regulated genes expression in Pseudomonas aeruginosa
Source: Front Microbiol. 2014 Nov 26;5:635. doi: 10.3389/fmicb.2014.00635 (PMC4244979; doi:10.3389/fmicb.2014.00635)

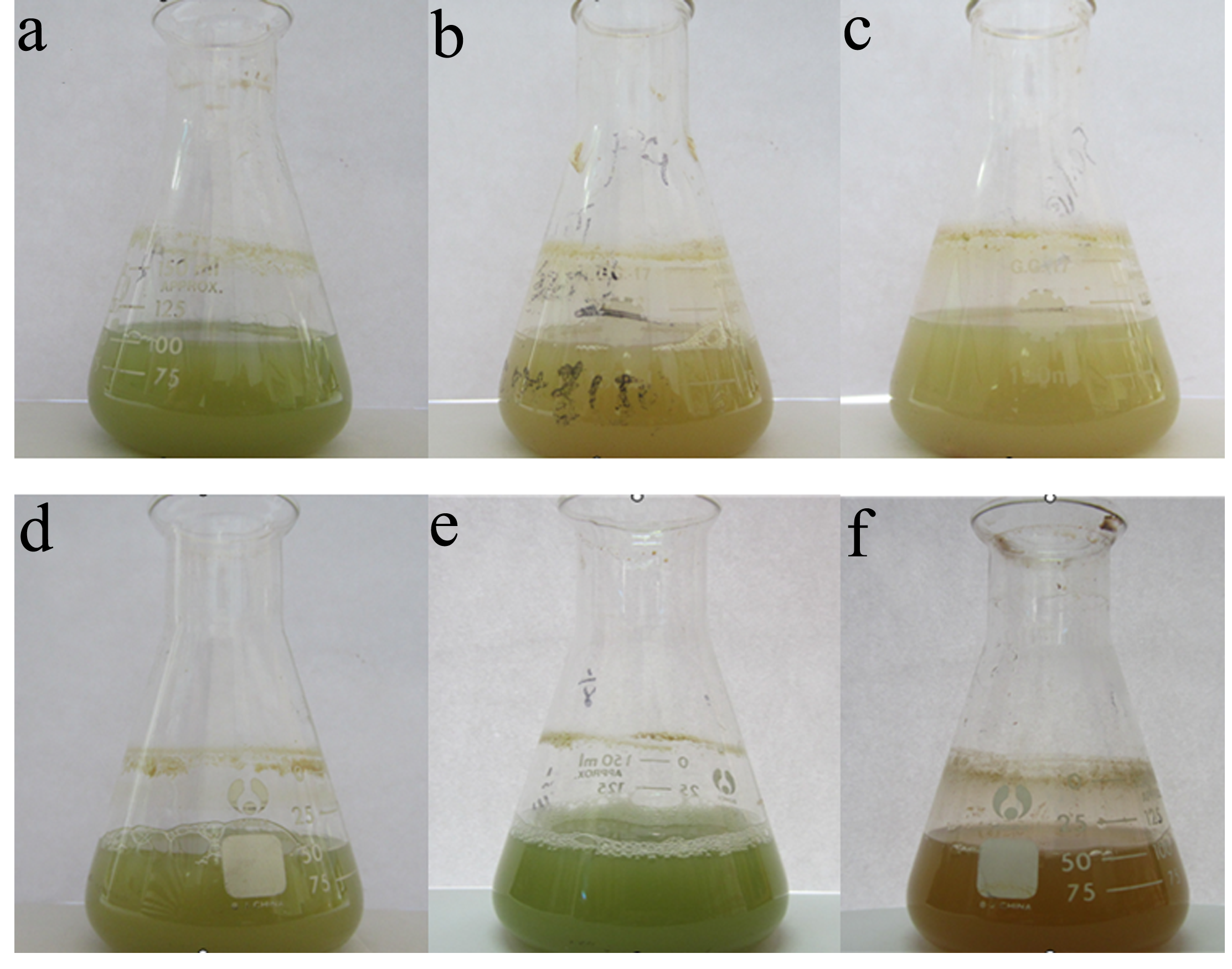

Supplement: Figure S1 — Color change of P. aeruginosa in response to SH. The color change of P. aeruginosa in liquid culture with various concentrations of SH [Control (without any drugs) (a), 64 μg/ml (1/8 × MIC) SH (b), 128 μg/ml (1/4 × MIC) SH (c), 256 μg/ml (1/2 × MIC) SH (d), 512 μg/ml (1 × MIC) SH (e) and 64 μg/ml (1 × MIC) AZM (f)]. [file Image1.TIF]

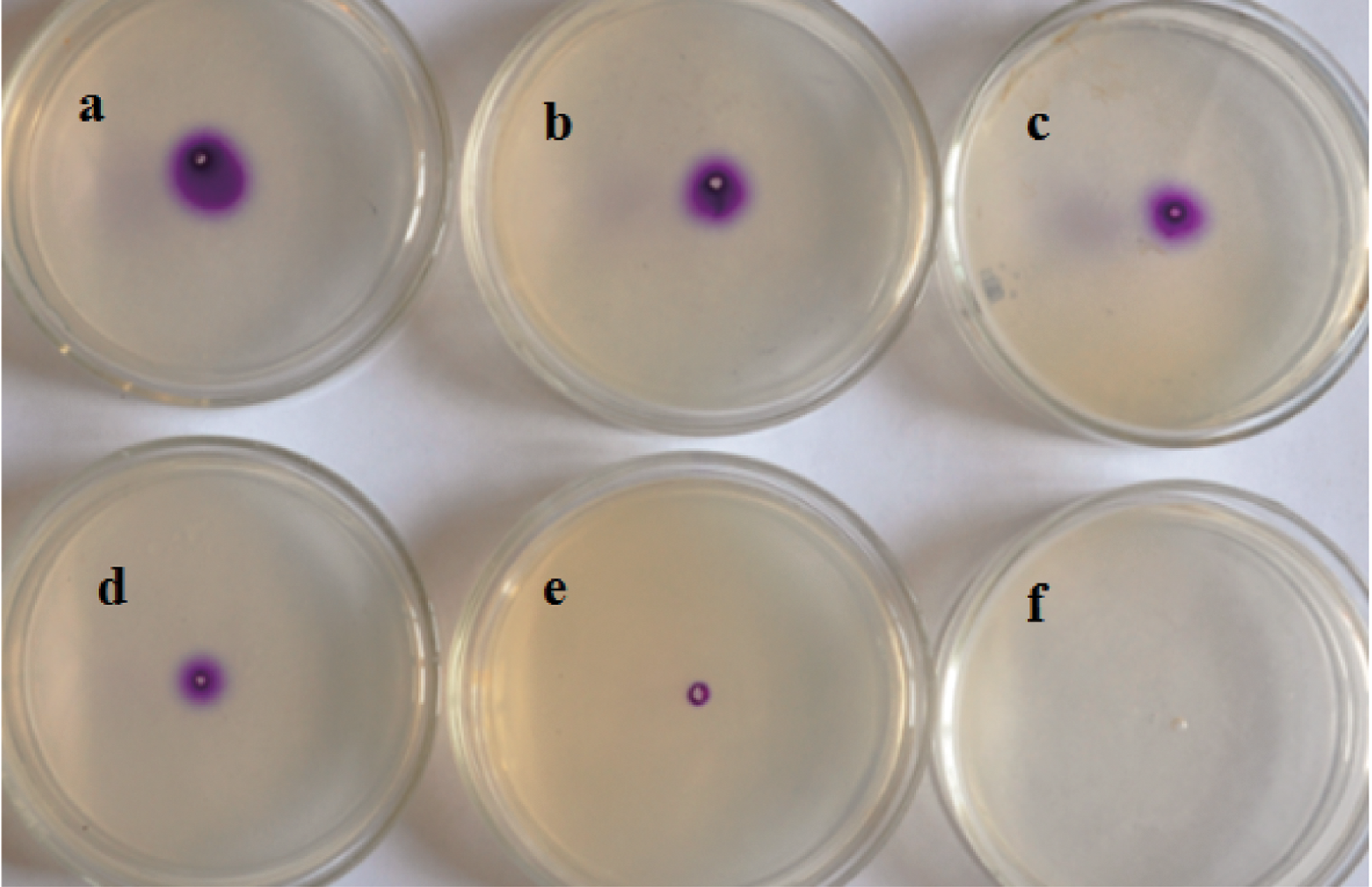

Supplement: Figure S2 — Plate assay demonstrating SH affecting AHL production. The AHLs from different groups of P. aeruginosa of Control (a), 64 μg/ml (1/8 × MIC) SH (b), 128 μg/ml (1/4 × MIC) SH (c), 256 μg/ml (1/2 × MIC) SH (d), 512 μg/ml (1 × MIC) SH (e) and 64 μg/ml (1 × MIC) AZM (f) were extracted and added into the center of CV026 plates. [file Image2.TIF]

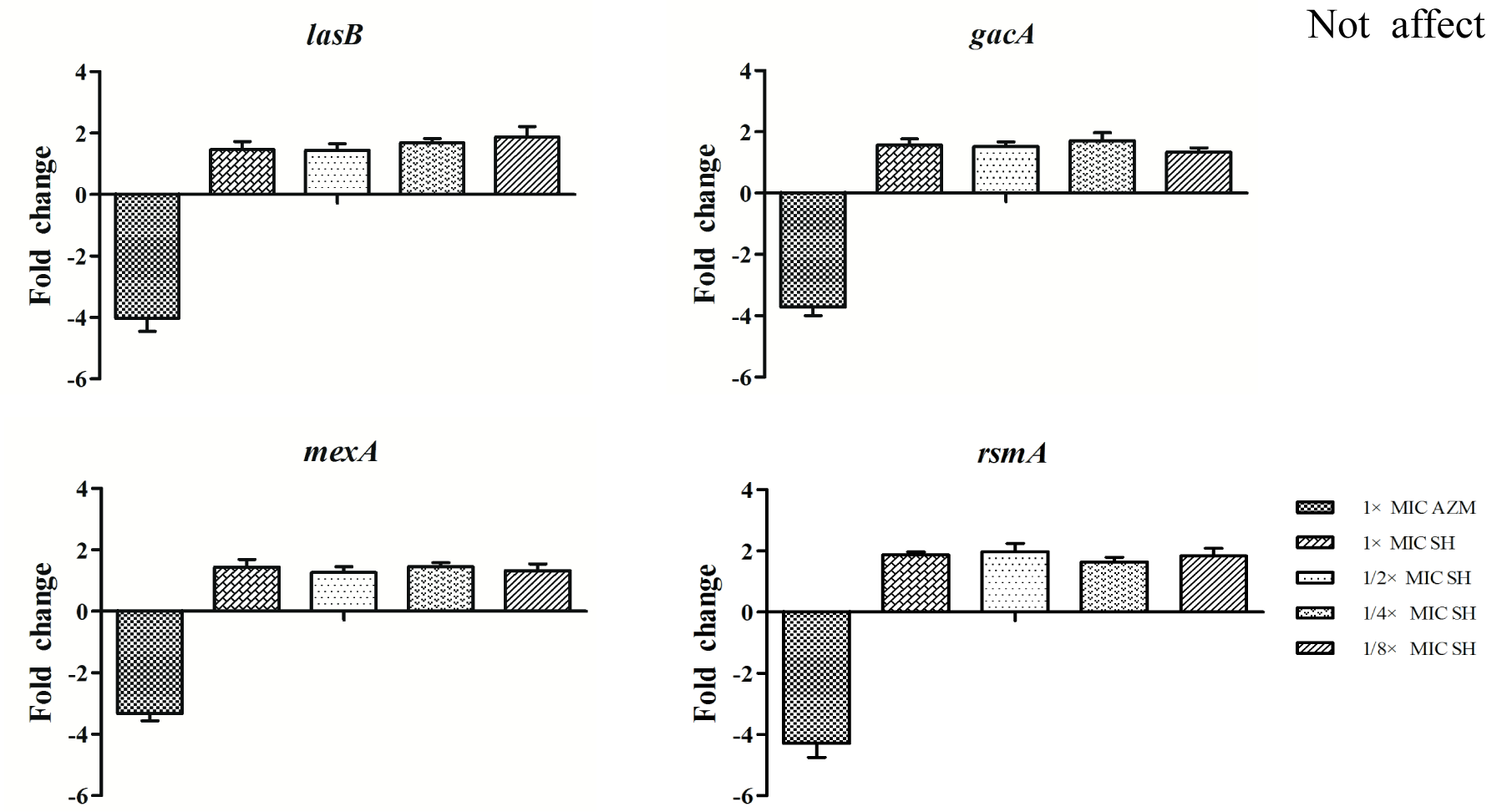

Supplement: Figure S3 — Effect of SH on the expression of lasB, gacA, rsmA and mexA. The expression of lasB, gacA, rsmA, and mexA were monitored in response to SH treatment. Expression of the house-keeping gene, rpoD, was used as the internal control for each sample. The drug concentration of treatments was as follows: 512 μg/ml (1 × MIC) SH, 256 μg/ml (1/2 × MIC) SH, 128 μg/ml (1/4 × MIC) SH, 64 μg/ml (1/8 × MIC) and 64 μg/ml (1 × MIC) AZM. [file Image3.TIF]
